# Feature combination networks for the interpretation of statistical machine learning models: application to Ames mutagenicity

**DOI:** 10.1186/1758-2946-6-8

**Published:** 2014-03-25

**Authors:** Samuel J Webb, Thierry Hanser, Brendan Howlin, Paul Krause, Jonathan D Vessey

**Affiliations:** 1 Lhasa Limited, Granary Wharf House, 2 Canal Wharf, Holbeck, Leeds LS11 5PY UK; 2 University of Surrey, Guildford, Surrey GU2 7XH, UK

**Keywords:** Interpretation, Interpretable, Machine learning, Mutagenicity, QSAR

## Abstract

**Background:**

A new algorithm has been developed to enable the interpretation of black box models. The developed algorithm is agnostic to learning algorithm and open to all structural based descriptors such as fragments, keys and hashed fingerprints. The algorithm has provided meaningful interpretation of Ames mutagenicity predictions from both random forest and support vector machine models built on a variety of structural fingerprints.

A fragmentation algorithm is utilised to investigate the model’s behaviour on specific substructures present in the query. An output is formulated summarising causes of activation and deactivation. The algorithm is able to identify multiple causes of activation or deactivation in addition to identifying localised deactivations where the prediction for the query is active overall. No loss in performance is seen as there is no change in the prediction; the interpretation is produced directly on the model’s behaviour for the specific query.

**Results:**

Models have been built using multiple learning algorithms including support vector machine and random forest. The models were built on public Ames mutagenicity data and a variety of fingerprint descriptors were used. These models produced a good performance in both internal and external validation with accuracies around 82%. The models were used to evaluate the interpretation algorithm. Interpretation was revealed that links closely with understood mechanisms for Ames mutagenicity.

**Conclusion:**

This methodology allows for a greater utilisation of the predictions made by black box models and can expedite further study based on the output for a (quantitative) structure activity model. Additionally the algorithm could be utilised for chemical dataset investigation and knowledge extraction/human SAR development.

## Background

(Quantitative) Structure Activity Relationships ((Q)SAR) models are widely applicable in drug discovery. With the large volumes of data available it is becoming easier to build models to predict biological activity and ADMET properties. There are three main methods for predicting the biological activity of compounds: grouping approaches such as read across, (Quantitative) Structure Activity Relationships ((Q)SARs) built using machine learning/statistical modelling and expert systems. All these methods rely on the similarity principle; similar structures exhibit similar activity [1].

A modeller using machine learning is spoiled for choice with regards to learning algorithm and descriptors for use in the development of predictive (Q)SAR models. The choices made can impact not only the predictive performance of the model but also the transparency of the prediction. If our goal is to make a model with the highest predictive performance possible we may choose a learning algorithm such as Random Forest (RF), Artificial Neural Network (ANN) or Support Vector Machine (SVM). These black box models (models with poor interpretability) will generally perform better on complex problems in comparison to white-box models (models with good interpretability) such as Decision Trees (DT) or Linear Regression (LR). Often a modeller will choose a trade-off between the performance of the model and the interpretability of the prediction according to the purpose of making a prediction. Further, the choice of descriptors will also impact on the interpretability of the model. However, even descriptors with a clear physical or chemical meaning will not adequately remove the black-box nature of models such as RF, ANN and SVM.

Models that do not allow for an interpretation of the cause behind the prediction can be underutilised as the user cannot easily assess the prediction. Models that facilitate the identification of the cause of the predictions provide richer support for structure optimisation stages. For example, consider a model that identifies a structure as mutagenic and in addition suggests the structural motif(s) that cause the prediction.

### (Q)SAR and knowledge mining

Research has been undertaken to mitigate this ‘black box’ issue of poor interpretability and trends in the literature are discussed here.

**Figure 1:**
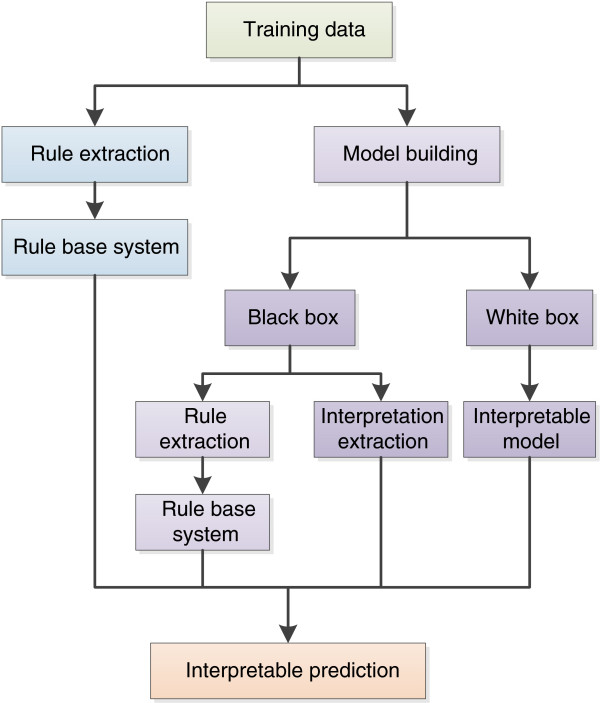
Knowledge mining and interpretation workflows.

Figure 1 represents various approaches to acquiring an interpretable prediction. Firstly we have rule extraction approaches directly from data which are utilised to produce a rule base predictive system. Alternatively we have approaches that rely on a machine learning model where we produce either an interpretable model or a poorly interpretable model (black box). We can either undertake rule extraction on the poorly interpretable model to produce a rule based system which provides interpretation or we can extract an interpretation from the model.

Knowledge mining approaches can be used to support the development of (Q)SAR models by human experts, facilitate descriptor selection (or generation) for models or to support the automated generation of interpretable rule bases for prediction. Methods for knowledge mining include emerging pattern mining (EP mining) [2,3], fragmentation [4], tree building through maximum common substructure analysis [5], rule extraction from models built from algorithms such as neural networks [6] and support vector machines [7]. A common issue with rule mining approaches is the loss in performance from the statistical model itself; information can be lost during the extraction step and the resultant interpretable models are frequently not as accurate as the original model resulting in a trade-off between rule clarity and accuracy [8]. However, another study has found that the rules extracted from a neural network can actually have a larger generalizability than the original model [9].

### Interpretation of predictions

Direct generation of an interpretable predictive model with no knowledge mining step relies on the model to provide a meaningful interpretation of the given prediction. The interpretation aims to ascribe particular features to the cause of the prediction made by the model. This cause (SAR) is acquired by defining the understanding of the model and its behaviour based on statistical relationships, and as such is a hypothetical but not necessarily an established chemical/biological SAR. These methods describe a likely “cause of the prediction” and will return why model X produced prediction Y, or support the user’s analysis of the relationship. This differs from a rule extraction approach in that a human expert may be able to remove rules that look erroneous (correlated, but not chemically meaningful) or even adjust rules based on identified local trends (context). This process would not be undertaken during this machine-based approach and as a result a prediction may be returned where an interpretation looks wrong to the expert user. It should be noted however that the interpretation method does not change the prediction outcome; it is adding on a new level by giving a cause of the prediction.

Some learning algorithms are able to give a global ranking of descriptors such as the Random Forest or partial least squares (PLS) algorithms. These measures are of global importance across a dataset, though they may already provide some insight into the model. However on a query by query basis this is a very coarse level interpretation. Guha *et al.* have shown that the Random Forest descriptor importance approach can also be used on artificial neural networks [10].

Another approach is to support the prediction with the visualisation of training structures. Stanton has reported success in developing SAR using PLS when using this interpretation approach [11]. Hansen *et al.* have developed a method to allow for the interpretation of models built using kernel based learning algorithms. The explanation of the model is provided by returning the most relevant structures to the prediction [12] providing a similar interpretation to that of Stanton.

Others such as Carlsson *et al.* have developed approaches to identify the most significant feature towards a prediction. They successfully used a decision gradient function from RF or SVM models to identify the most significant descriptor for a prediction. The decision function is analysed to determine the impact of each descriptor to the local neighbourhood and the descriptor with the largest gradient (impact) is identified. When coupled with fragment based toxicophore descriptors this has allowed for the identification of locally significant toxicophores for a given prediction [13].

Other approaches have been developed with the aim of assigning positive or negative contribution towards a prediction, i.e. atoms (x, y, z) cause a contribution towards active/high value and atoms (a, b, c) contribute towards inactive/low value. Kuz’min *et al.* have developed a methodology for determining atom contributions towards a regression prediction of a Random Forest model [14]. Ajmani *et al.* have developed a methodology for improving the interpretation of PLS. Their G-QSAR method improves the interpretability of the PLS models by using descriptors that are localised to specific features in addition to providing the ability to account for combinations/relationships between structural features [15]. However, a significant onus is still present for user input in providing the interpretation of the model. A similar approach has been developed by Guha *et al.*[16]. Baskin *et al.* have developed a methodology for producing an interpretation from artificial neural networks utilising the approach taken in methods such as linear regression [17].

Franke *et al.*[18] have developed a technique for identifying importance of potential pharmacophore points to the prediction of a query. Models are built based on fingerprints where the bits represent a potential pharmacophore point, bit importance is then acquired by measuring the change in prediction when a feature is removed [18]. Rinker and Landrum [19] have developed a similar approach for investigating fingerprint similarity and bit importance to a machine learning model. The work we present has a similar concept which has been extended to the investigation of the combination of bits within a fingerprint.

The early version of this work [20] produced an interpretation based on the impact of combinations of fragments present in the feature vector of a query structure. This work was then developed further to remove the dependency on descriptors with discrete structure based descriptors [21]. Since publication of this method Polishchuk *et al.*[22] published their approach of fragmenting the structure and defining the contribution of a fragment as the difference between the predicted value of the parent and the predicted value of the parent with the fragment removed. However, in this approach the interpretation will not be able to elucidate all of the information available on structures containing multiple toxicophores where the activity is binary. The removal of a single toxicophore may not change the prediction, which is a limitation acknowledged by the authors [22].

### Mutagenicity

Compounds can be mutagenic through a number of mechanisms. The most common is direct reaction with base-pairs of DNA for which the bacterial mutagenicity assay is well established [23]. Bacterial testing has a number of benefits including low cost, quick test time, straightforward test procedures and good correlation with lengthy rodent carcinogenicity studies [23]. The reverse mutation assay (Ames test [24]) is a common procedure involving the reverse mutation of histidine dependent *Salmonella typhimurium* and *Escherichia coli* strains. However, the testing procedure has multiple variants and with testing strategies dating back many decades the reproducibility of the results can suffer. Studies have been carried out investigating the reproducibility and quality of Ames test data finding that reproducibility ranges from 80-85% [25]. Factors including tested strains, concentration, choice of S9 (rodent enzyme) matrix and sample quality all affect the quality of the activity data [25].

The Ames mutagenicity assay produces a binary classification of mutagen/non mutagen of compounds for each test strain used; this allows an overall call to be made. It is known that the mutagenic potential of a chemical may be as a result of an active metabolite; which is simulated by the addition of rodent (commonly rat and hamster) metabolic activation (rodent S9 matrix) to provide a method for production of potentially mutagenic metabolites [23]. However, this is not a complete replication of mammalian in vivo conditions [26].

### Purpose of the work

The aim of the work presented here has been to develop an interpretation methodology for Ames mutagenicity prediction that is agnostic to the statistical machine learning algorithm used. The resulting algorithm should also be able to identify multiple causes for the presence or absence of activity. As far as possible the algorithm should be chemically meaningful, however fundamentally the interpretation is identifying the cause behind the prediction and not the cause of activity. Given a sufficiently well-built model, the cause of activity and the cause of prediction should be very similar and inferring from one to the other should become possible.

The developed algorithm breaks down the structure and assesses structural motifs for activity and relationships between motifs. The assessment can classify structural motifs of the query structures into different groups including activating, deactivated and deactivating. Localised deactivations can be identified and as a result a global active prediction can still produce an interpretation output containing a deactivated feature. It is also possible to identify multiple deactivations for the same structural component. The algorithm is currently optimised for endpoints such as a mutagenicity where the activity is heavily based on the presence of reactive groups and inactivity can be defined as the absence of mutagenic structural features.

## Algorithm

The developed algorithm allows for the extraction of relationships between the prediction and the patterns that the model is using to make it. The prediction remains that of the model and we supplement the prediction with the investigation of the model’s behaviour for a specific query structure. This investigation can be carried out directly on a feature vector if the vector represents a binary fingerprint of meaningful bits (approach 1). Alternatively fragmentation of the query structure prior to generation of the feature vector allows more freedom in descriptors (approach 2). In both cases we are evaluating the model’s behaviour on a subset of the query and mapping this to atoms and bonds present in the structure.

**Figure 2:**
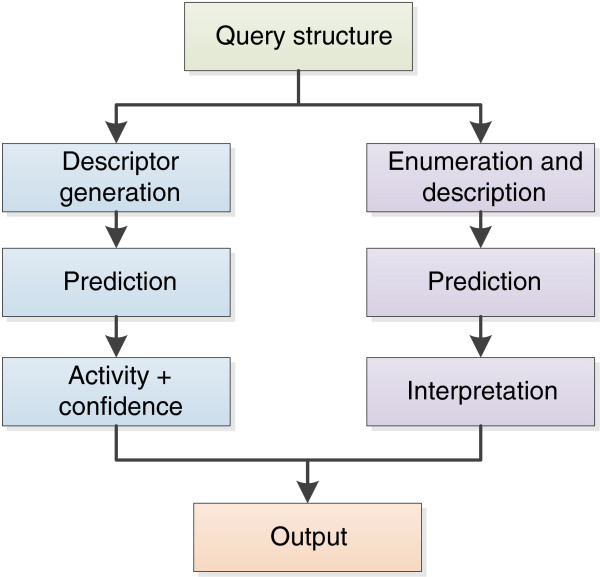
Stages for the generation of a prediction from a query structure.

The stages involved are shown in Figure 2 where we see the general prediction methodology and the additional route for the interpretation of a prediction.

The interpretation is achieved by investigating the model’s behaviour on either specific structural motifs or an enumeration of a feature vector. This allows the formation of a link between the outcome and the structural motifs present in the query.

To allow flexibility in the approach the methodology is separated into components, which themselves can be implemented in different ways: combination enumeration, network building and interpretation. A combination represents a subset of the features present in the query structure, the network represents the organisation of the enumerated combinations and the interpretation represents the extraction of the model’s behaviour and representation to the user.

### Combination enumeration

The interpretation aims to indicate the impact of structural features present in the query to the outcome of the model. This is achieved by investigating the model’s behaviour on the feature vector generated by the query structure. To do this we undertake combination enumeration on the feature, either by direct enumeration of the fingerprint itself (approach 1), or by fragmenting the structure and linking bits in the fingerprint to atoms and bonds in the query (approach 2).

Both approaches involve combination generation without repetition, i.e. a specific feature can only be present once in a given combination. This enumeration is represented in Equation 1 where n is the number of components and k is the desired number of components in the combination. Exhaustive enumeration is shown in Equation 2.

(1) $$C\left(n,r\right)={\;}^{n}C_{k}={\;}_{n}C_{k}=\frac{n!}{k!\left(n-k\right)!}$$

Equation 1 Combinations without repetition where n is the number of items and k is the desired number of items.

(2) $$C_{\mathrm{total}}=\sum_{i=0}^{n}C\left(n,i\right)=2^{n}$$

Equation 2 Total number of enumerable combinations where n is the total number of components (bits).

This combination enumeration is applied to the feature vector itself in approach 1 and to the structure in approach 2.

#### Approach 1: feature vector enumeration

The feature vector must be based on a binary fingerprint where a set bit represents the presence of a feature. To support the interpretation of a prediction these bits must also be meaningful e.g. represent distinct chemical substructures. This allows us to map the impact of the combination to specific atoms and bonds in the query.

**Figure 3:**
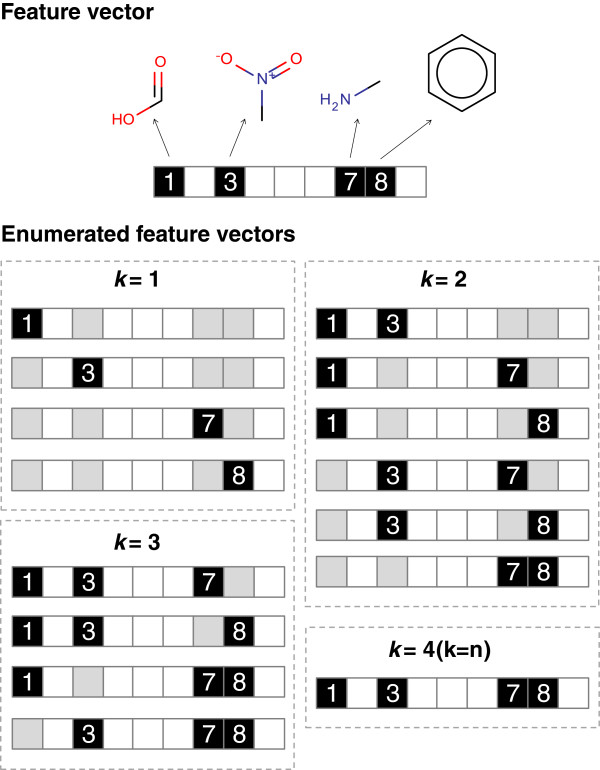
**Feature vector enumeration where k represents the number of bits to select for the combination.** A black box represents a set bit, a white box an unset bit and a grey box represents a bit set in the parent but not the enumeration.

In Figure 3 we see the results of the exhaustive enumeration of the combination {1, 3, 7, 8}, we have a total of 15 enumerations to process through the model. In this approach the enumerated combinations represent the feature vector to submit. The combination where k is equal to n represents the original query. This approach does not account for the connection between the features and can result in the identification of activations or deactivations from disconnected features.

#### Approach 2: structure enumeration

The second approach is to enumerate the structure rather than a feature vector directly. This approach broadens the scope of descriptor choice as we no longer need to identify the origin of a bit in a structural fingerprint. We can map the fingerprint bits to given atoms and bonds by generating the fingerprint for the fragment. For example this method opens up the interpretation to hashed fingerprints. However, not all descriptors are appropriate to calculate with this method; in practice the approach should be limited to structural features such as fingerprints and fragments.

**Figure 4:**
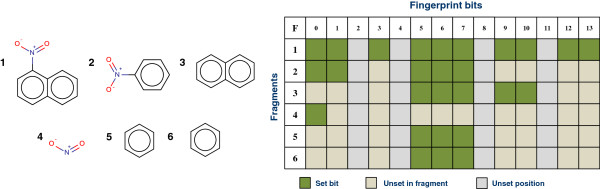
**Fragment enumeration (left) and theoretical description (right).** The bits set in the fingerprint represent the contribution of the fragments atoms and bonds to the parent structures fingerprint. A fragment will generate a subset of the bits set in the parent (or the full set).

In Figure 4 we see an example fragmentation of 1-nitronaphthalene where 6 fragments have been produced (fragment 1 being the query, 1-nitronaphthalene). Each fragment must be processed through the descriptor calculation methodology to generate the feature vector to then be processed through the model. The fingerprint generated for a fragment represents a subset of bits present in the fingerprint for the query structure. The figure shows how bits in a fingerprint can be linked to a structural motif on the query (parent) structure. For example bit 0 can be linked to the nitro group, for more complex relationships generating the fingerprint from the feature allows us to map the set bits to atoms and bonds on the source (parent) structure.

Unlike with the feature vector enumeration we do not have disconnected fragments due to the restrictions this poses on descriptor calculation. In the context of mutagenicity we also wish to limit the enumerations to connected combinations only.

### Feature networks

The feature network represents an organisation of the enumerated combinations. Traversing up the network represents an increase in the number of bits in a combination. The term ***node*** refers to a vertex of the network graph. In the context of the fragmentation based approach a node will contain a structural fragment, feature vector, identifying information and later a prediction and assessment.

**Figure 5:**
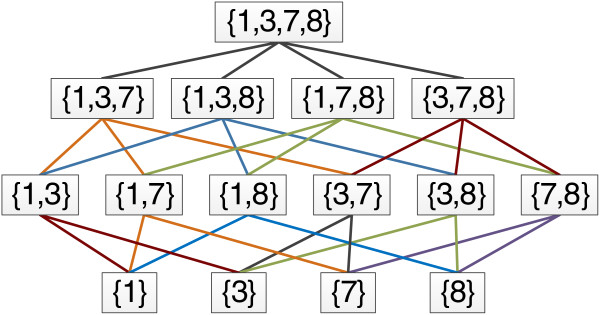
**Example feature network.** Parent feature represents the BitSet {1, 3, 7, 8}, all child nodes in the tree represent an enumerated combination. Decreasing the level in the network represents a decrement of 1 for the value or k.

A parent node represents a union of its children e.g. {1, 3, 7} is a union of {1, 3} and {1, 7}. A full network based on the example seen in Figure 3 is shown in Figure 5.

For approach 1 the parent child relationship can be identified by a subset-superset relationship and the level is identified by the k value. In the case of approach 2 the k level is discarded as this is highly dependent upon the type of fragmentation used. Instead the atom and bond ID’s can be used to organise the fragments into a network.

#### Limitations and practical implications

In both approaches we produce a network which can be traversed and visualised. We are able to project the results onto structural motifs on the query structure. In the case of direct descriptor enumeration this projection can take the form of disconnected features. However, in the case of the fragment networks disconnected features shouldn’t be produced due to the need for descriptor calculation.

The feature vector enumeration approach sufferers from computational intractability when the fingerprints are complex. To calculate the total number of combinations we can use Equation 2. When enumerating exhaustively the number of enumerated combinations is exponential with the increasing cardinality of the binary fingerprint.

**Figure 6:**
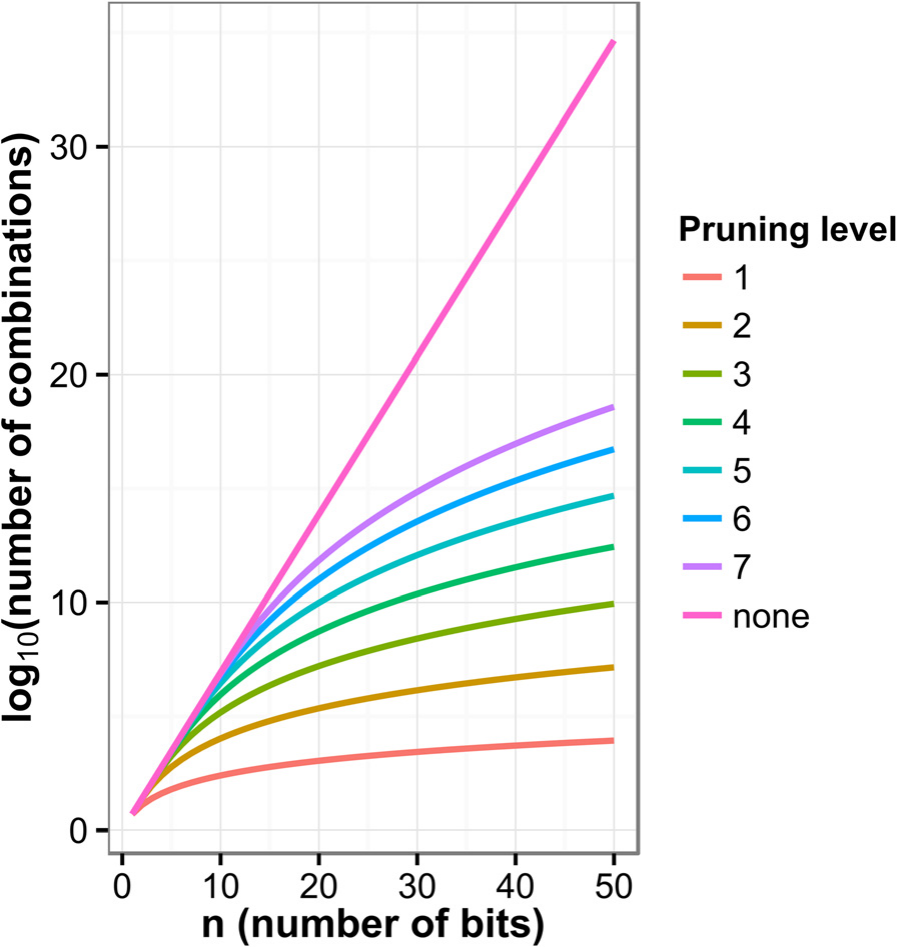
**Combination enumeration count with pruning.** Where the pruning level indicates the maximum k (number of bits) allowed for the enumeration and all levels below are included.

In Figure 6 we see that with exhaustive enumeration (pruning level = none) the number of nodes in the network becomes unmanageable as we increase the cardinality of the fingerprint. One approach to tackle this issue is to prune the enumeration step by limiting the number of elements in a combination. The figure shows the result of enumerating up to a specific number of elements for 1 through 7. This reduces the size of the network significantly but the impact of the network may vary depending on the endpoint and density of the fingerprint.

This issue is mitigated in the fragment network approach, although at the cost of the loss of disconnected fragment patterns. The fragment networks vary in size depending on the fragmentation algorithm used and further pruning could be undertaken if desired.

Both these approaches rely on the use of structural features as descriptors; the models discussed here are based on hashed fingerprints and structural keys. For fingerprint enumeration the bits in the fingerprint must be uniquely identifiable to allow to relationship between the bits and atoms and bonds on the query. In the case of the fragmentation, the fingerprints are generated on the fragments allowing for hashed fingerprint to be used. The fragment is used to map the bits to atoms and bonds, see Figure 4. This descriptor limitation is imposed in part by the treatment of a molecule as the sum of its parts and linking the impact of substructures of the query to the cause of the prediction. Utilising a model built on global properties such as logP and molecular weight would not able to be interrogated in the method describe here. By utilising only structural feature information in our descriptors (structural fingerprints/keys) the descriptor generation of a fragment results in a subset of features with regards to the parent structure. In this way we are mapping the models behaviour on the fingerprint subset to the structural feature(s) on the query responsible for their generation.

The algorithm described here is applicable to binary endpoints where a class boundary of active/inactive can be defined. Given the limitation of descriptor choice, endpoints that can be described by the contribution of structural motifs are best suited. The models will not be capturing global molecular properties that aren’t described by the fingerprint.

### Network assessment and summary

The organisation into a network facilitates the implementation of a number of assessment and summary approaches; we discuss the method developed for the assessment of Ames mutagenicity here. For mutagenicity the following criteria governing activity have been assumed:

1) The activity of a compound can be described by the presence of a structural feature.

2) The inactivity of a compound can be described by:

a. The lack of an activating feature.

b. The deactivation of all activating features.

**Figure 7:**
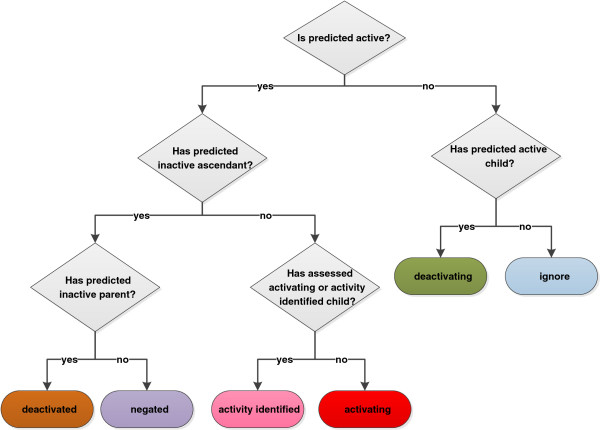
Node classification rules represented as a decision tree.

Although these feature networks allow for direct navigation a method of summarisation has been developed to provide a succinct and meaningful explanation of the model’s behaviour for a given query. An algorithm was developed to classify each node in the network as {*ACTIVATING, DEACTIVATED, DEACTIVATING, NEGATED, ACTIVITY_IDENTIFIED, IGNORE*}. These classifications are explained in Table 1 and the rules are given in Figure 7.

**Table 1:** Assessment rules

**Type**	**Description**
ACTIVATING	ACTIVATING nodes are the first occasion in the network path (starting from the bottom) where an active feature has been found and is not deactivated. An activating node can have descendant nodes that are predicted active if their assessed type is not activating (i.e. the node has been deactivated or negated).
DEACTIVATED	A DEACTIVATED node is one in which the predicted class is active but the node has an inactive parent. Deactivated nodes can be deactivated by multiple parents.
DEACTIVATING	A DEACTIVATING assignment occurs when a child node is predicted active but the current node is predicted inactive. The class has switched from active to inactive so a deactivation has occurred. A deactivating node only deactivates children, not more remote descendants.
NEGATED	A NEGATED node is one in which the predicted activity is active, all parents are predicted active but at least one ascendant is inactive. The node is not set to deactivated as a deactivating node can only deactivate a child, thus defining the specific contextual relationship of the deactivation which is a superset of the negated component.
ACTIVITY_IDENTIFIED	A node is classified as ACTIVITY_IDENTIFIED when it has an activating descendant. Activity is assigned to the lowest feature in the path not the highest.
IGNORE	A node is set to IGNORE when it is predicted inactive and has no impact on the nodes below it.

In the relationships a node can be deactivated by multiple parents and a deactivation can likewise deactivate multiple children. When making an assessment both the predicted class and the assessed type of other nodes may be accounted for.

**Figure 8:**
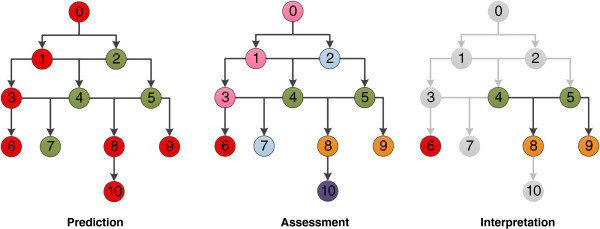
**Network example.** The prediction network is coloured according to activity (red = active, green = inactive), the assessment network is coloured according to assessment type (red = activating, pink = activity identified, blue = ignore, green = deactivating, orange = deactivated, purple = negated). Finally the interpretation network is shown with only the nodes of interest coloured (according to the network assessment scheme).

To illustrate the algorithm let us consider the example network in Figure 8 which provides an example of every assessment type.

The left network is coloured according to the prediction provided by the model, for each fragment the network represents a red node as an active prediction and a green node as an inactive prediction. The middle network is coloured according to the assessment of each node where red is activating, green is deactivating, orange is deactivated, blue is ignore, purple is negated and pink is activity identified.

Let us consider each node independently; **node 6** has no children and only active ascendants (3, 1, 0). The fragment in this node results in an active prediction and the model does not consider any larger context of the fragment to be inactive. We can therefore assign the node to be activating and identify it as an independent cause of an active prediction. **Node 7** has no children and is inactive, we need not consider this node further and assign it to ignore. **Node 10** is predicted active, has an active parent but it has 3 inactive ascendants (2, 4, 5). Node 10 is not directly deactivated but the activity is lost further up the network so it is set to negated, this is a subset of a larger deactivation context. **Nodes 8 and 9** are predicted active but have only inactive parents and as a result deactivated as they are not sufficient to cause an active prediction. **Nodes 4 and 5** are predicted inactive and have predicted active children so they are deactivating of an active feature. **Node 2** is inactive, has no assessed active descendants (as the predicted active nodes have been deactivated) and is therefore set to ignore. Finally **nodes 0, 1 and 3** are all predicted active but are ascendants of an assessed active node at position 6. These nodes are therefore set to activity identified; they are still predicted active but the context of the fragment at node 6 was sufficient alone for the active prediction.

A summary of the network is then undertaken to allow for succinct reporting of the cause of the prediction, the nodes involved in the summary are shown in the right network of Figure 8. This takes the form of a list of activations and deactivations present in the network. In this example this summary would be of the form: 6, 4–8, 5–8 and 5–9 where each number represents a node. A feature network must not contain any activating nodes for a final prediction to be active. However, an active prediction can have any number of deactivations as long as there are 1 or more activating nodes.

We therefore have the following potential scenarios in a summary output:

1) Atom(s) {a, b, c} and bond(s) {x, y} are deemed to be ACTIVATING

2) Atom(s) {a, b, c} and bond(s) {x, y} are deemed to be DEACTIVATED, by atom(s) {d, e} and bond(s) {z}

The output can therefore be thought of as identifying the atoms and bonds without which the predicted class may switch. For example a prediction stating that atoms {1, 2, 3} and bonds {1, 2, 3} are identified as ACTIVATING with no other relationships found would identify that the removal of those atoms and bonds would result in the query structure no longer being considered active. Likewise removal of the atoms present in the DEACTIVATING component of a deactivation would result in a cause of a positive prediction.

## Experimental methods

### Software

The KNIME workflow package [27] has been utilised for data processing, model building and prediction as well as the framework for the development of the prototype methodology. A Java API has been written to add additional modelling and processing functionality.

Additionally, our in-house chemical engine has been incorporated into KNIME by a number of new nodes and cell types covering a variety of cheminformatic techniques. Some of these features can also be found in existing KNIME plugins from RDKit [28] and CDK [29] but to provide the most control we utilised our own code wherever possible. Standardizer and Structure checker from ChemAxon [30] were used in combination with our engine to identify and then curate issues in the data. Models and results in this paper are generated using the KNIME modelling nodes; however any modelling package could be used.

### Data preparation and curation

A curation effort was undertaken to improve the quality of the structural data and briefly assess the potential reliability of the experimental results of a mutagenicity benchmark dataset (Hansen) that was constructed by combining data from multiple sources [31]; not all of these sources provide the data in a readily available format. CCRIS [32] and GENETOX [33] data are provided in a web interface with structures being represented in a picture format. Another limitation is caused by the lack of a unique identifier common between the source and benchmark datasets. The combination of ChemAxon software and various cheminformatic KNIME nodes allowed for an easy identification of issues and a semi-automated curation procedure. Curation was only undertaken on structures; the activity remains that of the initial dataset. However, a simple comparison where CAS numbers are known and comparable to the original dataset shows the experimental activity to be the same.

Data were acquired from the following data sources, Hansen [31], Bursi [34], NISS [35], Helma [36], GENETOX [33], CCRIS [32], CPDB [37] and Vitic Nexus [38]. A curation of the benchmark data was then undertaken using in the following approach:

1) Where original source data were deemed of higher quality replace the benchmark structure where the structures are readily available

2) Replace all known benchmark structures with Vitic structures (match by CAS)

3) Treat mixtures: remove salts, remove structures containing significant multiple components (such as CAS 3546-41-6, Pyrvinium pamoate)

4) Remove structures containing X or R atoms

5) Identify and fix structural issues such as misrepresentation of nitro groups

6) Clean and redraw the structures including aromatization and removal of explicit hydrogens and stereochemistry

7) Check experimental activity is consistent between the various data sources

8) Remove duplicates

This public curated data was then split into a large training set of 5297 structures and a randomly selected test set of 1325 structures.

### Performance measurement

A number of metrics are available for the assessment of predictive performance. The models here are binary classifiers and the following measures have been utilised to assess the predictive performance of the models based upon true positive (TP), false positive (FP), true negative (TN) and false negative (FN) result classification. The measures are: area under ROC curve (AUC), balanced accuracy (BAC), sensitivity (SENS), specificity (SPEC) and coverage (COV), see Table 2.

**Table 2:** Performance measures used

**Name**	**Equation**	**Usage**
Balanced accuracy (BAC)	$\frac{\mathrm{SEN}+\mathrm{SPEC}}{2}$	Accuracy measure that accounts for bias in the data.
Sensitivity (SEN)	$\frac{\mathrm{TP}}{\mathrm{TP}+\mathrm{FN}}$	Model’s ability to correctly identify positives
Specificity (SPEC)	$\frac{\mathrm{TN}}{\mathrm{TN}+\mathrm{FP}}$	Model’s ability to correctly identify negatives
Coverage (COV)	$\frac{\text{in}\;\text{domain}\;\text{structures}}{\text{Number}\;\text{of}\;\mathrm{structures}}*100$	Model’s coverage of the given validation set representing the applicability of the model.

5 fold cross validation (CV) has been utilised to estimate the generalisation error of the model. The validation sets were determined randomly and assigned prior to model building. Therefore the same splits have been used on all of learning algorithms. The folds are split with 1059 structures in folds 1–4 and 1061 structures in fold 5. Area under the curve (AUC) has also been used as a measure incorporating the confidence of the model as well as the predicted performance [39].

### Learning algorithms

Optimisation of each learning algorithm was undertaken based on cross validation results. For decision tree models those built with pruning produced models of higher accuracy than the unpruned trees on the same descriptor set. For kNN an unweighted approach was utilised and a generally optimal k value of 8 was found from investigation on internal validation trends. The Tree Ensemble learner was used and configured in such a way to produce a variation of Random Forest. Previous experience on this data has shown that the split criterion of Information Gain Ratio produced better models than information gain or Gini index. No minimum node size or depth was set, the fraction of data in the bag was set to 0.7 without replacement. Attribute sampling was set to the square root of the number of attributes and a new sample taken at each node. For the final forest model 200 trees were built.

LibSVM version 2.89 is implemented within KNIME v2.7.3 and available through the update site. For SVM models the learner and predictor nodes available were utilised using the C-SVC SVM and the Radial Basis Function (RBF) kernel. The grid search algorithm provided with LibSVM v3.17 was utilised for the optimisation of the cost (C) and gamma (γ/g) parameters of the RBF kernel used for learning. This optimisation was undertaken outside KNIME. The grid searching algorithm explores the parameter space defined and the defaults of log_2_C (−5, 15, 2) and log_2_γ (3, −15, −2) were used.

### Descriptor calculations

The four types of structural fingerprints available in the KNIME CDK fingerprints node have been used for model building: MACCS, CDK standard, CDK extended and Pubchem all provided by CDK [29]. Mol blocks were converted to CDK structures, fingerprints were generated and the fingerprint was then expanded and appended to the structural information. Additionally our in house atom centred fingerprint was generated using our chemical engine. Again, the fingerprint was expanded into Integer values where 0 = not present, 1 = present.

The MACCS and Pubchem fingerprints are based on structural keys where each bit denotes a specific piece of information such as an unsaturated 5 membered ring or a specific SMARTS pattern. The CDK fingerprints and our own are hashed fingerprints where a specific bit cannot be traced back to a specific chemical feature. The standard CDK fingerprint ignores cyclic systems whereas the extended CDK fingerprint considers them [29]; further information can be found in the CDK Javadoc.

### Applicability domain

To facilitate comparison between the algorithms and descriptors an applicability domain methodology that is agnostic to descriptor choice and learning algorithm was chosen. The fragmentation based domain [40] is a simple method of domain assignment where all fragments on the query being present in the training set results in an ‘in domain’ result and new fragments on the query result in an ‘out of domain’ result. The fragmentation algorithm used is able to discover larger contexts around a structural motif and a slightly adapted methodology was taken. Step 1: fragment the training set and store the dictionary if the fragment occurs 4 or more times. Step 2: for each query structure generate constituent fragments and check for the presence in the dictionary. If the fragment is in the dictionary remove the corresponding atoms and bonds from the unmatched list. If any atom or bond remains once all fragments have been processed then the structure is outside of the domain of the model.

### Fragmentation

Rather than fragment the original molecule, the fragmentation method first builds an intermediate reduced graph where all the nodes represent a structural unit of the original molecule. The scope of a structural unit is flexible and can be adjusted to different use-cases. Structural units can for instance represent single atoms and bonds, functional groups, rings, fused rings, etc. Once the reduced graph has been constructed we fragment the reduced graph using a combination of circular and linear path enumerations. Finally each fragment generated from the reduced graph is expanded back to a molecular fragment graph. The depth of the path enumeration can be configured. This fragmentation method allows us to take advantage of an exhaustive path enumeration without the risk of breaking the use-case related logical units within the molecules.

**Figure 9:**
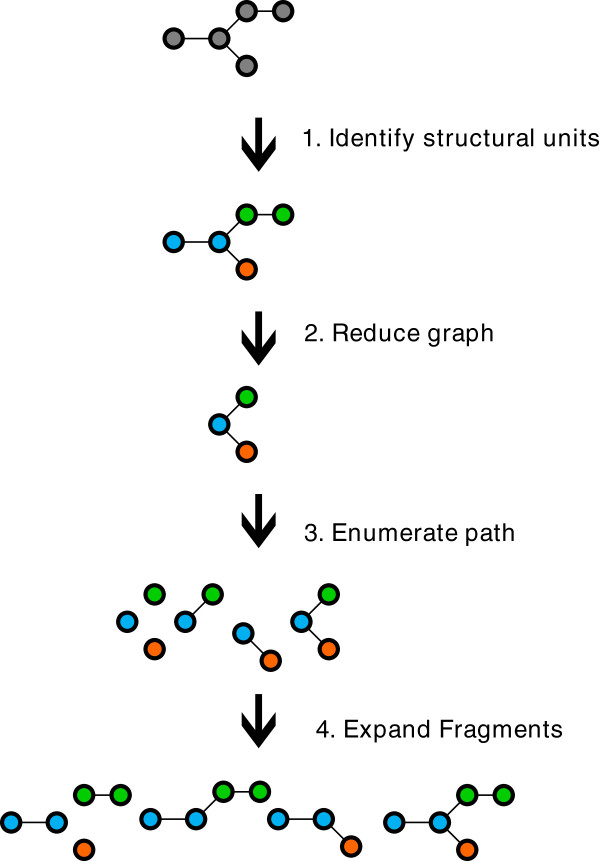
Reduced graph fragmentation.

This approach is shown in Figure 9. However any fragmentation approach could be implemented that allows for a hierarchy to be built.

### Interpretation

The interpretation was implemented with a Java component and access to it was provided as a KNIME node. The node accounts for the required network building and searching and provides as output the summary and a network view allowing for manual interaction with the fragment network. Each node in the fragment network stores: the fragment; prediction; confidence (if provided); atom and bond id’s of parent compound; index position; parent index; child index; ascendant indices; and, descendant indices. This information is utilised in the network search and assessment.

An example KNIME workflow is provided in the Additional file 1 with example outputs and network visualisation.

## Results and discussion

Here we discuss the performance of the learned models from cross validation and against external validation sets before discussing the interpretations produced against a selection of the validation data.

5-fold cross validation shows that the SVM models are stable across the different descriptor sets whereas the other modelling algorithms are susceptible to a change in descriptors. Pubchem fingerprints produced the most accurate models regardless of learning algorithm used. On average the models have a balanced accuracy c. 80% for SVM and RF and 75% for DT and kNN. Full details can be found in the Additional file 1. The black box approaches of the SVM and RF have a clearly better performance than the DT and kNN models.

For this dataset the SVM models have a similar accuracy to the RF models; the balance between sensitivity and specificity of the models differs, however. The RF models have a bias towards sensitivity at the cost of specificity, whereas this bias is not so pronounced in the SVM models. The DT and kNN models also have a bias towards sensitivity. They however have poor specificity. The endpoint is driven by the presence of mutagenic features and the DT and kNN models appear to be unable to pick up on the subtleties causing inactivity on structures containing potentially mutagenic features. The results from the cross validation therefore show that strongly predictive black box models should be used as the relationships are too complex for the more interpretable models like DT and kNN to produce equivalent performance. Previously a desire for interpretability may have played a factor in choosing a lower performing model, however our method of interpretation allows us the benefit of a wider range of learning algorithms for Ames mutagenicity prediction.

The structural key based fingerprints (MACCS and Pubchem) show a higher performance than the hashed fingerprints (CDK standard and extended); however, as a whole the descriptor choice is not the significant factor in the model performance. It is therefore likely that the information encoded in the keys is able to better describe in good detail the features behind the mutagenicity of the structures. However identification of information that falls outside that encoded by these keys will not be possible by the models.

### External validation performance

Each model was used to predict a random external validation set of 1325 structures of which 1282 are classed as in domain by the fragment based domain approach (97% coverage).

Again Pubchem descriptor based models as a whole produced better performance than those built from other fingerprints. As with the cross validation studies the models have a biased performance towards sensitivity and again the difference is more pronounced in the RF model than the SVM models. We can see from Table 3 that the DT and kNN models only fall 2-6% short of the sensitivity of the SVM and RF models. However the specificities are much lower with a loss of 3-11% depending on model and descriptor choice.

**Table 3:** Publc validation set performance for all models and descriptor sets

												
	**MACCS**				**Pubchem**				**CDK standard**			
	**AUC**	**BAC**	**SEN**	**SPEC**	**AUC**	**BAC**	**SEN**	**SPEC**	**AUC**	**BAC**	**SEN**	**SPEC**
SVM	0.87	0.81	0.82	0.79	0.88	0.82	0.84	0.80	0.87	0.81	0.84	0.78
RF	0.88	0.82	0.86	0.77	0.88	0.81	0.86	0.76	0.88	0.81	0.83	0.79
DT	0.81	0.77	0.80	0.74	0.79	0.76	0.78	0.74	0.80	0.75	0.79	0.72
kNN	0.84	0.76	0.84	0.68	0.84	0.77	0.81	0.73	0.83	0.75	0.81	0.70
	**CDK Extended**				**Atom centered**							
	**AUC**	**BAC**	**SEN**	**SPEC**	**AUC**	**BAC**	**SEN**	**SPEC**				
SVM	0.87	0.81	0.83	0.79	0.88	0.82	0.84	0.80				
RF	0.87	0.80	0.82	0.78	0.88	0.81	0.82	0.80				
DT	0.78	0.75	0.80	0.71	0.79	0.75	0.79	0.71				
kNN	0.84	0.77	0.81	0.73	0.84	0.77	0.82	0.72				

AUC = area under curve, BAC = balanced accuracy, SEN = sensitivity, SPEC = specificity.

Aromatic amines (primary, secondary and tertiary) cover 16% of the training set and aromatic nitro compounds 13% with some overlap between the two sets. These features impose a significant bias on the learning and validation. The external validation set has been broken down into specific regions of chemical space (not accounting for co-occurrence of the features) and details of these regions can be found in Table 4.

**Table 4:** Specific region of chemical space training and validation distribution

**Label**	**Feature**	**Training**		**Validation**	
		**Count**	**Active bias**^**a**^	**Count**	**Active bias**
a	Aromatic amine (primary)	573	0.72	117	0.67
b	Aromatic amine (secondary)	113	0.61	28	0.61
c	Aromatic amine (tertiary)	168	0.60	38	0.63
d	Aromatic nitro	736	0.85	206	0.81
--^b^	Aziridine	39	0.95	13	1.00
e	Epoxide	248	0.75	62	0.61
f	Carboxylic acid	425	0.29	109	0.32
g	Aliphatic halogen	534	0.65	149	0.62
h	Bay-region polycylic hydrocarbon	190	0.86	39	0.87

a = % of compounds in set with active experimental class, b No negative examples in the validation set.

**Figure 10:**
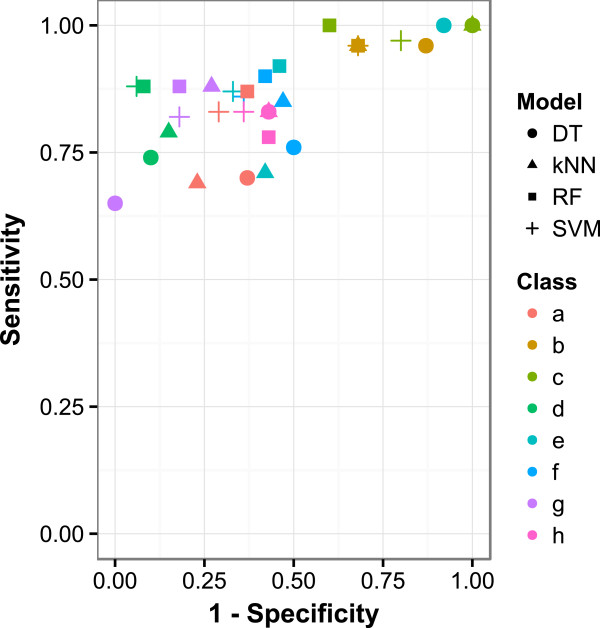
ROC plot of PubChem descriptor model performance for split chemical space validation set.

The SVM and RF models perform consistently well with regards to sensitivity across these subsets. Both the kNN and DT models struggle particularly with secondary aromatic amines, epoxides, carboxylic acids, and structures containing aliphatic halogens. The results of the best performing descriptor set (Pubchem) are given in Table 5 and visualised in Figure 10.

**Table 5:** PubChem descriptor model performance for split chemical space validation sets

**Algorithm**	**Measure**	**Aliphatic halogen**	**Aromatic nitro**	**Aziridine**	**Bay region PAH**	**Carboxylic acid**	**Epoxide**	**Aromatic amine (primary)**	**Aromatic amine (secondary)**	**Aromatic amine (tertiary)**
SVM	**AUC**	0.83	0.78	---	0.73	0.96	0.85	0.82	0.91	0.77
	**BAC**	0.77	0.64	NaN	0.58	0.91	0.77	0.75	0.82	0.73
	**SEN**	0.83	0.96	1.00	0.97	0.88	0.87	0.86	0.82	0.83
	**SPEC**	0.71	0.32	NaN	0.20	0.94	0.67	0.64	0.82	0.64
RF	**AUC**	0.8	0.8	---	0.72	0.94	0.87	0.84	0.95	0.82
	**BAC**	0.75	0.64	NaN	0.70	0.9	0.73	0.74	0.85	0.68
	**SEN**	0.87	0.96	1.00	1.00	0.88	0.92	0.9	0.88	0.78
	**SPEC**	0.63	0.32	NaN	0.40	0.92	0.54	0.58	0.82	0.57
DT	**AUC**	0.66	0.58	---	0.65	0.87	0.56	0.71	0.82	0.57
	**BAC**	0.66	0.55	NaN	0.50	0.82	0.54	0.63	0.82	0.70
	**SEN**	0.7	0.96	0.92	1.0	0.74	1.00	0.76	0.65	0.83
	**SPEC**	0.63	0.13	NaN	0.00	0.9	0.08	0.50	1.00	0.57
kNN	**AUC**	0.77	0.79	---	0.74	0.91	0.70	0.78	0.88	0.78
	**BAC**	0.73	0.64	NaN	0.5	0.82	0.65	0.69	0.8	0.70
	**SEN**	0.69	0.96	1.00	1.00	0.79	0.71	0.85	0.88	0.83
	**SPEC**	0.77	0.32	NaN	0.00	0.85	0.58	0.53	0.73	0.57

Where NaN = not a number result as all predictions were true positive, AUC = area under curve, BAC = balanced accuracy, SEN = sensitivity, SPEC = specificity.

With regards to specificity the performance of the kNN model is closer to that of the SVM and RF models, however DT still falls short. The DT model shows a significant failure to capture inactivity in aromatic nitro and epoxide containing structures. All models struggle to capture the inactivity of some primary aromatic amines, tertiary aromatic amines, aromatic nitro, epoxides and bay region containing polycyclic aromatic hydrocarbons. Likely causes are the poor representation of the inactive structures containing these motifs. A local modelling approach for these strongly activating features would likely produce better predictions for these regions of chemical space although more data will still likely be required. This issue may also be as a result of the descriptor choice which is limited to structural fragments/features.

### Interpretation

Here we discuss some example interpretations and the differences between the various RF and SVM models.

#### Example 1 (with network)

**Figure 11:**
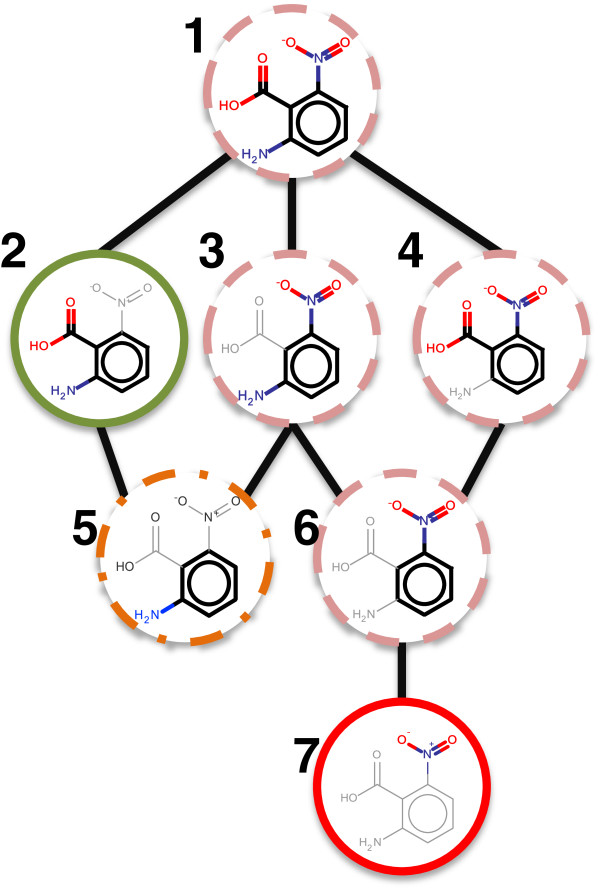
Example interpreted network where the nodes are coloured as: red (solid) = ACTIVATING, pink (dashed) = ACTIVITY IDENTIFIED, orange (dot – dash) = DEACTIVATED, green (solid) = DEACTIVATING.

First let us consider the network for 2-amino-6-nitrobenzoic acid which illustrates a real prediction with a localised deactivation on a globally predicted active structure. The model used for interpretation is the SVM built using Pubchem fingerprints. For clarity the nodes classified as ‘ignore’ are not shown and constitute benzene, the carboxylic acid and the amine group (all of which were predicted inactive by the model). The illustrated network can be seen in Figure 11.

The network shows that the model considers the aromatic amine fragment (node 5) to be active based on statistical evidence in the dataset. However, with the addition of the ortho position carboxylic acid the model predicts inactive. Here we have identified a deactivation of the aromatic amine moiety by the carboxylic acid. Independent of this relationship the algorithm has identified that the model perceived the nitro to be active (node 7). This activity is carried up the network through nodes 1, 3, 4 and 6 which have therefore been assigned as ACTIVITY_IDENTIFIED. As a result the summary output for this network consists of the nitro motif activation and the deactivation of the aromatic amine. Investigation of the network itself facilitates a deeper understanding of the relationships and the confidence values associated with each node. The summary however allows the condensation of the network of 8 nodes into two highlighted structures where the activation is represented by the highlight of the nitro in red, the second structure highlight would be represented by an orange aromatic amine and a green carboxylic acid. Such a scheme is shown in the following figures.

#### Example 2

2-(1-Naphthylmethyl)oxirane is an experimentally active structure in the Ames mutagenicity assay and contains the mutagenic epoxide toxicophore.

**Figure 12:**
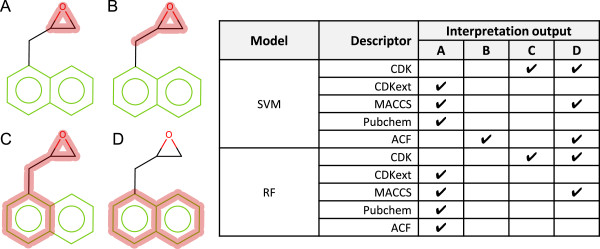
**2-(1-Naphthylmethyl)oxirane interpretation for RF and SVM models.** A red highlight denotes an ACTIVATING feature. **A-D** refer to a specific highlight summary produced by the models shown in the table.

Figure 12 shows the interpretation of each SVM and RF model. The pubchem and CDK extended models have identified the epoxide fragment as the only cause of the active prediction. The CDK standard models have not been able to identify the epoxide fragment in a localised context, likely due to ignorance of cyclical systems. In addition the naphthalene scaffold fragment has also been identified as a cause of the active prediction. The MACCS key active prediction has been identified to be caused by the epoxide (in its most local context) and the naphthalene scaffold fragment. Our atom centred fingerprint resulted in the identification of the epoxide in both models. The RF model also identifies the naphthalene scaffold as an ACTIVATING feature.

The epoxide fragment occurs 248 times in the training set and 185 (75%) of the occurrences are in experimentally active structures. The naphthalene scaffold fragment occurs 772 times with 623 (81%) of the occurrences being experimentally active. The naphthalene fragment is a substructure of many polycyclic aromatic hydrocarbons, many of which are mutagenic in their own right. Naphthalene is also experimentally inactive in the Ames mutagenicity assay [41]. We can conclude from these results that although the models may learn that the naphthalene moiety is active this is a statistical correlation and not a chemical one.

#### Example 3

**Figure 13:**
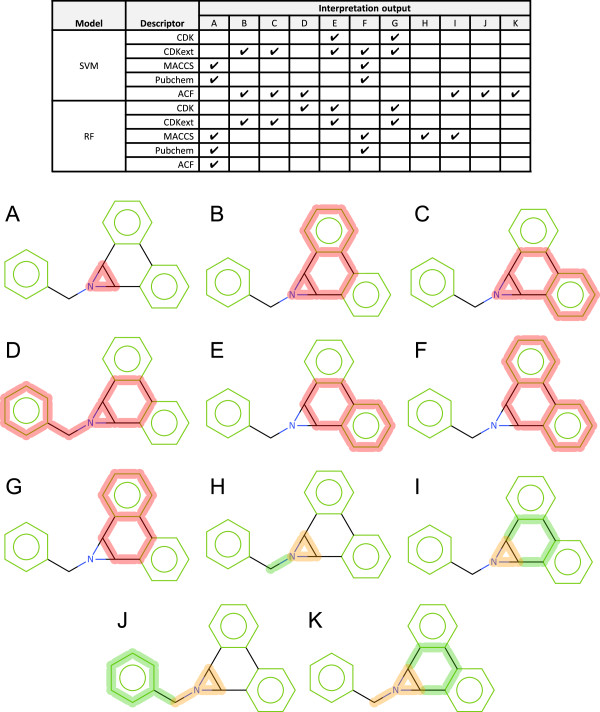
**1-Benzyl-1a,9b-dihydro-1H-phenanthro[9,10-b]azirene interpretation.** A red highlight denotes an ACTIVATING feature, a combination highlight is encoded with orange representing DEACTIVATED atoms and bonds and green representing DEACTIVATING atoms and bon. **A-K** refer to a specific highlight summary produced by the models shown in the table.

1-Benzyl-1a,9b-dihydro-1H-phenanthro [9,10]-b azirene is experimentally active for Ames mutagenicity. Each model correctly predicts this structure as active. However, as we can see from Figure 13 the cause of the prediction differs between models.

The training set contains 6 analogues of this query structure with various substitutions on the single benzene ring, of these 5 are mutagenic. The occasion that this is not the case the structure has significant changes with an addition fused ring system and a tertiary amine.

The aziridine scaffold moiety is a known mutagenic toxicophore and is present in rule base systems such as Derek Nexus. Therefore the Pubchem and MACCS model identification of this feature can be seen as a successful identification of a mutagenic feature by the model. The interpretation of the CDK fingerprint does not produce an interpretation localised to the aziridine moiety, standard + SVM misses the feature, standard + RF finds it in a larger context, extended + SVM again finds it in a larger context and finally the extended + RF model has found a deactivation of the aziridine moiety and moved to a larger context. Similar behaviour is seen with our atom centred fingerprint; however, the SVM + ACF identifies the aziridine motif in the smallest context. This behaviour highlights a limitation in the descriptor set; the models have not identified the activity of the aziridine moiety when described by the CDK fingerprints. In the case of the standard fingerprint this is not surprising as cyclic systems are ignored. The training set contains 39 structures with the aziridine moiety of which 37 are active.

Additionally activity is seen relating to the 9,10-dihydrophenanthrene ring scaffold. Analysis of the training set reveals 54 structures containing this substructure of which 46 are experimentally active. Further analysis of this set of structures reveals that of the 46 experimentally active structures 42 have at least one toxicophore such as aziridine, epoxide or aromatic nitro. It is likely that the activity of this fragment is a statistical artefact of co-occurrence with other mutagenic features and not as a result of being a mutagenic feature itself.

#### Example 4

1-Ethyl-2-Nitrobenzene is reported as experimentally inactive in the Ames assay and has the aromatic nitro toxicophore present.

**Figure 14:**
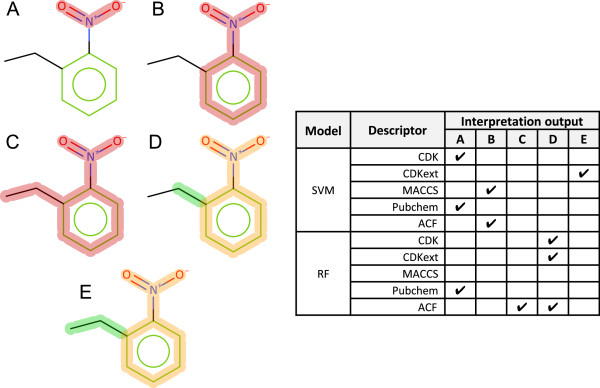
**1-Ethyl-2-Nitrobenzene interpretation for SVM and RF models.** A red highlight denotes an ACTIVATING feature, a combination highlight is encoded with orange representing DEACTIVATED atoms and bonds and green representing DEACTIVATING atoms and bonds. **A-E** refer to a specific highlight summary produced by the models shown in the table.

Of the models represented in Figure 14, the CDK standard RF, CDK extended RF and CDK extended SVM models and ACF RFwere able to identify the deactivation of the aromatic nitro toxicophore. In 3 cases the nitro fragment alone was sufficient to cause an active prediction regardless of the aromatic ring connection. Searching the training set for examples containing a nitro-benzene with a ortho substitution to the nitro substitution reveals 18 examples. 9 of the examples are active and of the 9, 4 examples have potential secondary toxicophores. Statistical analysis indicates that an ortho methyl substitution may be deactivating to the aromatic nitro toxicophore. In the atom centred SVM model the deactivation is not identified. In the atom centred RF model a deactivation is seen with the single carbon substitution, however the two carbon substitution is believed to be active by the model. The larger context has overridden the localised deactivation.

### Limitations in the interpretation: impact of fragmentation

The fragmentation methodology allows us to map subsets of the feature vector to atoms and bonds on the query providing a meaningful and simple visualisation of the elucidated interpretation. This fragmentation limits the both positively and negatively the search space generated. For example by not breaking open rings we remove any fragments that would be generate from partial ring features. This is a requirement for the descriptor generation; we cannot meaningfully produce the fingerprints on fragments containing query atoms. The knock on effect is that we may miss some of the generalisation of the model. If feature X connected to an aromatic carbon is sufficient to cause a positive prediction our interpretation would identify this as feature X connected to ring Y where ring Y contains the aromatic carbon.

How we identify ‘functions’ in our reduced structures also impacts on the elucidated interpretation. As with the aromatic carbon vs full ring mentioned above, the cause of the active prediction for the model may be a substructure in a reduced element. We cannot identify any smaller moiety than the atoms and bonds in a single reduced component. We would therefore assign the activity to additional atoms and bonds present in the smallest fragment containing the cause.

### Identified ‘toxicophore’ fragments

This interpretation algorithm has knowledge of the type of endpoint injected into the assessment algorithm. We consider the first node in a path predicted positive (and no change in activity in any ascendant) to be the root cause of the activity. This is meaningful for reactivity based endpoints based on the presence and absence of features.

During a cross validation study utilising our in house atom centred fingerprint and a Weka Random Forest model we can record the assessment of each node in the fragment based networks. This allows us to identify the features that have been deemed ACTIVATING by the model + interpretation combination. Each fragment has a unique identifier and details of the assessment, occurrence and accuracy of the model when the feature is present can be recorded.

**Figure 15:**
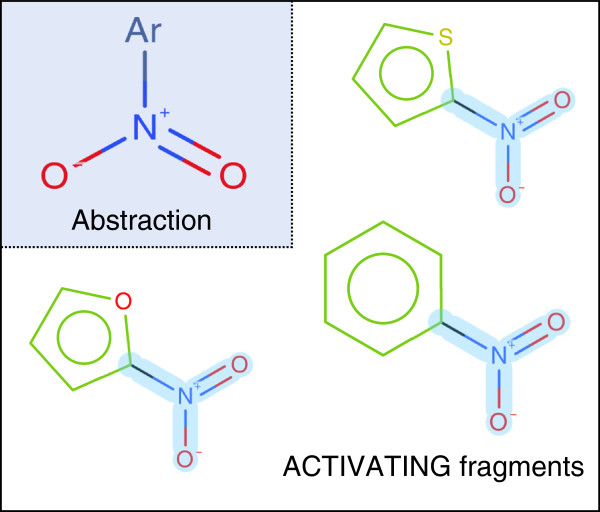
**Example of a difference in models learning and interpretations elucidation.** A model may learn that a pattern of aromatic nitro is activating. However, we are not able to generate a fragment describing this. As a result we would identify the activity as being caused by the aromatic nitro and the attached ring. The abstracted pattern is highlighted in blue on the ACTIVATING fragments.

From the training set, 210 ACTIVATING features were identified with an occurrence (number of structures containing the feature) > 5 and an assessment type of ACTIVATING > 5. Some features are independent of each other while others correlate strongly but form different substitutions or different ring types. For example nitrofuran, nitrothiophene and nitro benzene motifs are identified as separate entities, all containing the core aromatic nitro motif, see Figure 15.

**Figure 16:**
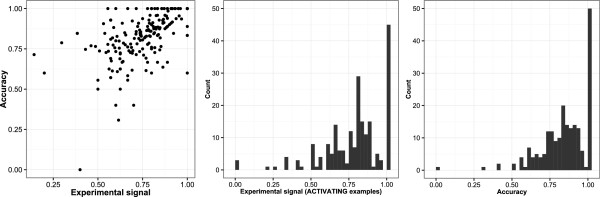
**ACTIVATING feature profiles.** Left, accuracy vs experimental signal (full supporting examples) of 210 ACTIVATING features. Middle, experimental signal (ACTIVATING examples only) histogram of the 210 ACTIVATING features. Right, accuracy histogram of the 210 ACTIVATING features.

In Figure 16 we see that the accuracy of the ACTIVATING features predominates around the accuracy of the model as a whole (c. 80%); there is a bias at experimental signal of 1 for where features are found containing only active examples, either due to the data or correct identification of the deactivations/exclusions. Some features have an experimental signal with a bias towards inactive structures (<0.5), however the model remains accurate in most cases. In other cases the model is shown to have misidentified a cause of activity.

**Figure 17:**
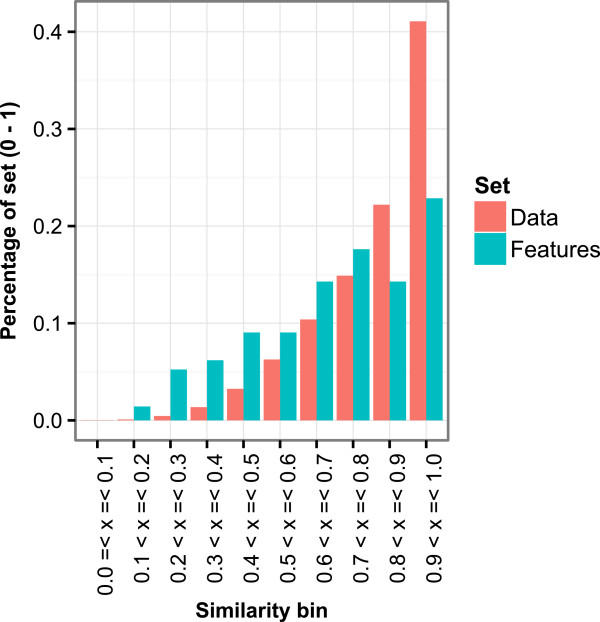
Maximum Tanimoto similarity of the ACTIVATING features and training data.

The average Tanimoto similarity of a 1 vs all comparison using our in house atom centred fingerprint (Figure 17) gives a value of 0.164 for the training data and 0.137 for the extracted ACTIVATING features, the activating features are therefore more diverse than the training set as a whole. 55% of the features have a similarity to another feature > 0.7. Where substitutions are important similar features will be generated for the various substitution patterns which cannot be described in abstract terms using this approach. Further, if the pattern described in the model is a functional group feature connected to any ring atom, this approach will always include the specific ring identified when assessing the structure. Out of the 210 extracted features 33 represent functional group motifs, 56 ring motifs and 121 a combination of functional group motifs and ring motifs. The 210 fragments with occurrence and performance metrics are included in the Additional file 1.

### Comparison with kazius toxicophores

Kazius *et al.*[34] derived a series of toxicophores for mutagenicity from a dataset of 4337 structures identifying a total of 29 toxicophores, 19 general and 10 additional. The approved toxicophores in the general group had their SMARTS patterns transcribed form the Additional file 1. The polycylic aromatic system SMARTS have been removed due to the authors’ stated limitations in describing the toxicophore with SMARTS. The remaining 18 toxicophores were compared with the 210 identified by our cross validation feature extraction approach.

**Figure 18:**
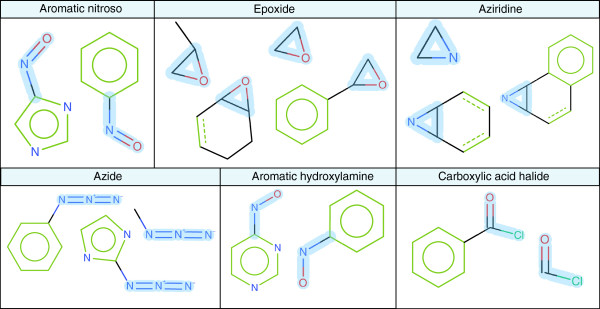
**Example Kazius toxicophore matches against ACTIVATING features.** Example comparisons of the Kazius general toxicophores and extracting ACTIVATING features, the Kazius toxicophore is highlighted on the fragment in blue.

The SMARTS patterns were used to filter the 210 ACTIVATING features to identify features that contained the Kazius toxicophore. 16 of the Kazius toxicophores have at least 1 corresponding ACTIVATING feature in our study, see Table 6. The aromatic azo toxicophore does not match, but has a similar feature described by a benzene ring connected to the diazo group, however the opposite ring connection is unspecified and therefore does not match this toxicophore from a SMARTS matching perspective. A corresponding ACTIVATING feature for the aliphatic diazo was not found. 93 of the 210 ACTIVATING features contained at least one of the Kazius general toxicophores. The Kazius toxicophores have a level of abstraction that is not replicated in our methodology; we therefore identify a variety of ACTIVATING causes around a central motif, see Figure 15 and Figure 18.

**Table 6:** Comparison of Kazius toxicophores with extracted ACTIVATING features

**Toxicophore**		**Signal**		**Performance**		
**Kaizus name**	**Count features**	**Full**	**Activating**	**BAC**	**SEN**	**SPEC**
Specific aromatic nitro	15	0.84	0.88	?	0.95	?
Specific aromatic amine	27	0.77	0.82	?	0.90	?
Aromatic nitroso	2	0.89	0.89	?	1.00	?
Alkyl nitrite	1	0.80	0.67	?	1.00	?
Nitrosamine	9	0.90	0.90	?	0.98	?
Epoxide	4	0.65	0.66	0.65	0.83	0.46
Aziridine	3	0.84	0.84	0.46	0.93	0.00
Azide	3	0.85	0.89	?	0.99	?
Triazene	1	0.78	0.88	0.75	1.00	0.50
Unsubstituted heteroatom-bonded-heteroatom	8	0.83	0.76	?	0.89	?
Aromatic hydroxylamine	2	0.79	0.77	0.50	1.00	0.00
Aliphatic halide	22	0.76	0.82		0.85	
Carboxylic acid halide	2	0.86	0.94	0.44	0.88	0.00
Nitrogen sulphur or mustard	1	1.00	1.00	?	1.00	?
Bay-region in Polycyclic aromatic hydrocarbons	1	0.80	0.82	0.64	0.94	0.34
K-region in Polycyclic aromatic hydrocarbons	1	0.80	0.82	0.64	0.94	0.34
Diazo	0	?	?	?	?	?
Aromatic azo	0	?	?	?	?	?

? refers to a value that could not be calculated.

#### Algorithm performance

The addition of interpretation inherently increases the time taken to process a query structure and two additional steps are added to generate the interpretation. The code has not been optimised for speed and is only single threaded within the KNIME framework, so one can expect significant performance enhancements in a production version of the system. However, to provide an indication of performance the following measurements were taken using a Windows 7 64-bit machine with an Intel® Core™2 Quad CPU Q9400 @ 2.66Ghz with a Java heap space of 6GB.

300 query structures were randomly sampled and a time footprint investigated. To process 300 through descriptor generation (fingerprint) and prediction requires 2625 milliseconds providing an average of 8.75 milliseconds per prediction. Running all predictions in sequence with interpretation the total time for prediction is 899656 milliseconds (15 minutes) with an average of 3000 milliseconds per structure. This is a significant increase in time. However 3 seconds per structure is within an acceptable timeframe for our needs. With more efficient processing the speed could be significantly increased. The network searching itself isn’t easily parallelisable. However the job of batch processing is and does not need to be processed sequentially as it is now.

## Conclusion

In this article we presented an interpretation algorithm able to provide meaningful interpretations of predictions from both Random Forest and Support Vector Machine models. The interpretations reveal interesting trends within the data, support further mining of the dataset seeded by highlighted features and allow the user to understand the differences between models built from different descriptors.

Where the networks produced are not complex it is possible to visually assess and investigate the behaviour of the model further than the summary results provided in the form of highlighted structures. This facilitates understanding of how the model perceives the increasing structural context around a feature; colour coding is also possible according to the confidence in the prediction of each node.

The algorithm can provide verbose output with regards to deactivations, especially where the molecules exhibit symmetrical features. The networks can also result in a sequence of deactivations. This issue can be addressed by keeping the largest context of a deactivation. For example a ring may be deactivated by a substituent resulting in the activity passing up the network only to be deactivated higher in the path. The deactivation at the highest point would be selected for representation of the behaviour.

This new approach is able to identify multiple activations and/or deactivations as well as localised deactivations where the final prediction is active. The algorithm requires no conversion step between a trained model and a rule set where a loss in predictive capability will occur. When coupled with a suitable cheminformatics platform the approach also supports further exploration of the chemical space based on the interpreted output of the model. This is achieved independently of the learning algorithm used.

This approach can allow an expert to quickly understand the reason behind a model’s prediction and the user to effectively dismiss predictions which although statistically correct, do not stand up to scientific scrutiny that has previously not been possible for users of black box systems.

The variations in substitution pattern and how explicit a feature becomes are issues that would need to be addressed for knowledge mining purposes. However, the algorithm has been developed for the interpretation of the models prediction rather than toxicophore mining. An iterative process while recording the fragment assessments already provides a strong basis for knowledge mining of toxicophores utilising statistical learning algorithms and this interpretation.

## Abbreviations

RF: Random forest; SVM: Support vector machine; ANN: Artificial neural network; (Q)SAR: (Quantitative) structure activity relationships; LR: Linear regression; DT: Decision tree; kNN: k Nearest neighbours; PLS: Partial least squares; ACF: Atom centred fingerprint.

## Competing interests

The authors declare that they have no competing interests.

## Authors’ contributions

SW: Undertook data curation. Development of KNIME nodes and workflows, development of interpretation algorithm and API, undertook model building, drafted the manuscript. TH: Developed the fragmentation algorithm, supported development of the interpretation algorithm. BH: Supported development of the interpretation algorithm. PK: Supported development of the interpretation algorithm. JV: Undertook data curation. Developer of supporting KNIME nodes, supported with the Java implementation of the network building and supported development of the interpretation algorithm. All approved the final manuscript.

## Supplementary Material

Additional file 1 **The following additional data are available with the online version of this paper.** Additional file 1 is providing graphics representing the KNIME implementation, pseudo-code for the assessment of the networks and additional validation results for the models. Click here for file
