# Supplementary material for: Feature combination networks for the interpretation of statistical machine learning models: application to Ames mutagenicity
Source: J Cheminform. 2014 Mar 25;6:8. doi: 10.1186/1758-2946-6-8 (PMC3997921; doi:10.1186/1758-2946-6-8)
Supplement: Additional file 1 — The following additional data are available with the online version of this paper. Additional file 1 is providing graphics representing the KNIME implementation, pseudo-code for the assessment of the networks and additional validation results for the models. [file 1758-2946-6-8-S1.pdf]

Supplementary information

KNIME single query interpretation workflow

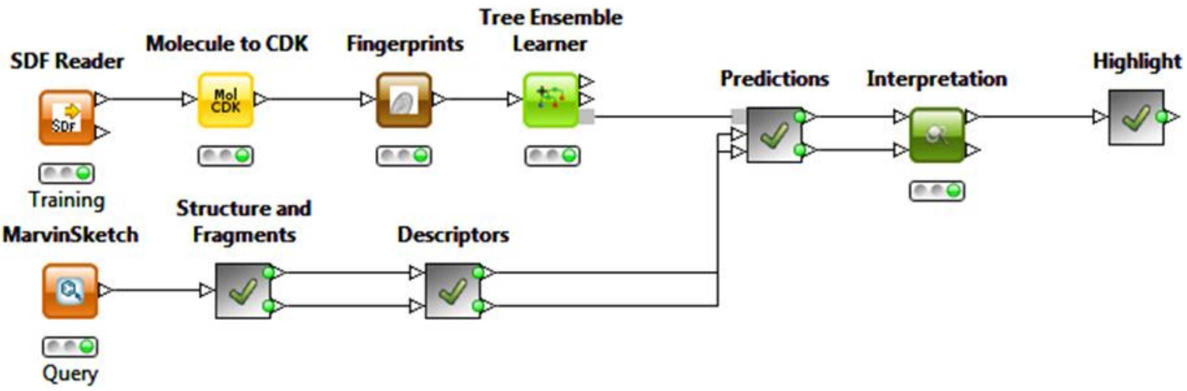

Meta nodes represent interchangeable components covering the titled functionality.

Interpretation node view

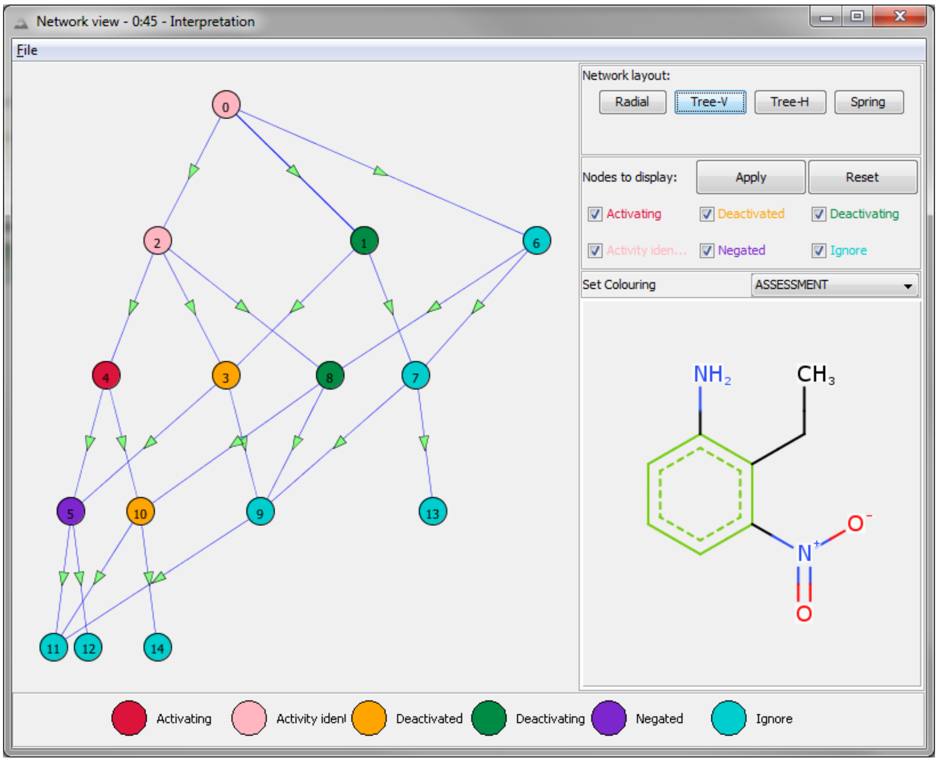

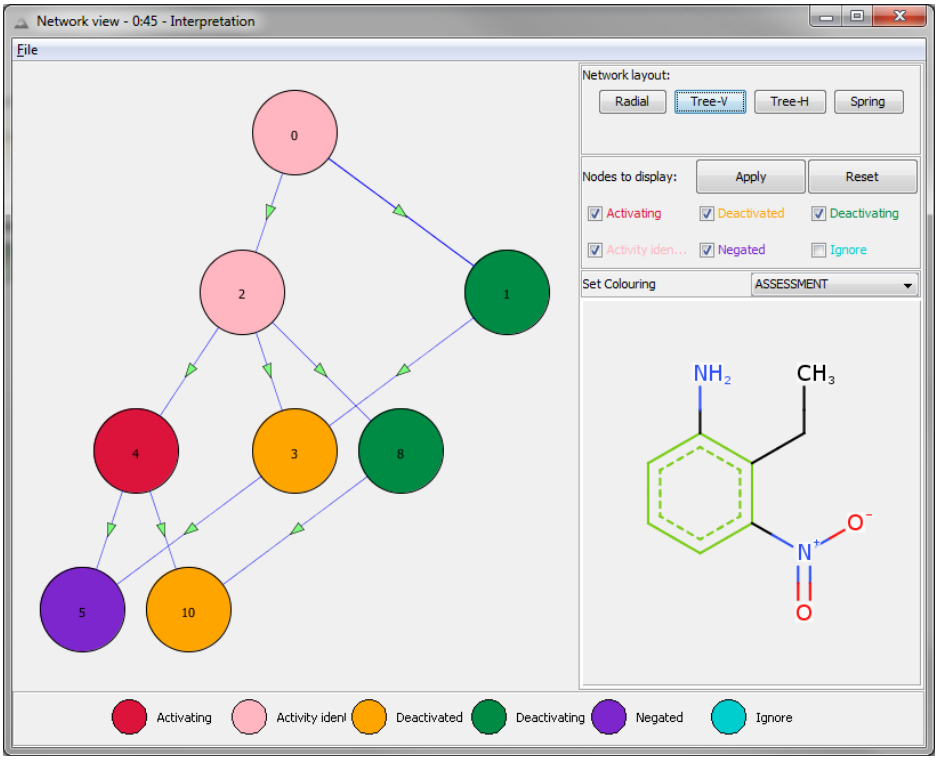

Example network for 2-ethyl-3-nitroaniline: upper image represents the full network drawn using the vertical tree layout from the Zest plugin and the lower image represents a filtered draw where nodes classified as ignore are not shown. Nodes are coloured by their assessment type; nodes can also be coloured by confidence in the prediction.

Pseudo code for network assessment

```
assesNode(rootNode)
{
    if(node has child)
    {
        for(each child)
        {
            // All the children must be assessed
            if(child not assessed)
                assessNode(childNodeId);
        }

        if(predicted activity == ACTIVE)
        {
            if(hasPredictedInactiveAscendant)
            {
                // DEACTIVATED if a parent is predicted inactive
                assessment = hasPredictedInactiveParent ? DEACTIVATED : NEGATED;
            } else
            {
                // ACTIVATING only if a child isn't assessed as ACTIVATING or ACTIVITY IDENTIFIED
                assessment = hasActivatingOrActivityIdentifiedChild ? ACTIVITY_IDENTIFIED : ACTIVATING;
            }

        } else
        {
            // DEACTIVATING only if a child is predicted active
            assessment = hasPredictedActiveChild ? DEACTIVATING : IGNORE;
        }

    } else
    {
        // For nodes with no children
        if(predicted activity == ACTIVE)
        {
            if(hasPredictedInactiveAscendant)
            {
                // DEACTIVATED if parent is predicted inactive otherwise negated
                assessment = hasPredictedInactiveParent ? DEACTIVATED : NEGATED;
            } else
            {
                // ACTIVATING as no loss of activity in path to the root
                assessment = ACTIVATING;
            }

        } else
        {
            assessment = IGNORE;
        }
    }
}
```

Cross validation results

Algorithm	Descriptors	AUC	BAC	SEN	SPEC
SVM	MACCS	0.86	0.80	0.82	0.78
	Pubchem	0.88	0.80	0.82	0.78
	CDK Standard	0.86	0.80	0.82	0.78
	CDK Extended	0.87	0.80	0.82	0.78
	ACF	0.87	0.80	0.82	0.77
RF	MACCS	0.88	0.81	0.83	0.78
	Pubchem	0.88	0.81	0.85	0.76
	CDK Standard	0.86	0.79	0.82	0.77
	CDK Extended	0.87	0.80	0.81	0.78
	ACF	0.87	0.79	0.80	0.78
DT	MACCS	0.79	0.76	0.79	0.72
	Pubchem	0.80	0.77	0.79	0.74
	CDK Standard	0.76	0.74	0.77	0.71
	CDK Extended	0.77	0.74	0.78	0.71
	ACF	0.78	0.74	0.79	0.71
kNN	MACCS	0.84	0.75	0.84	0.66
	Pubchem	0.83	0.76	0.82	0.69
	CDK Standard	0.82	0.75	0.82	0.68
	CDK Extended	0.83	0.75	0.81	0.70
	ACF	0.83	0.75	0.79	0.71

AUC = area under curve, BAC = balanced accuracy, SEN = sensitivity, SPEC = specificity
